# A Multi-Objective Approach for Optimal Energy Management in Smart Home Using the Reinforcement Learning

**DOI:** 10.3390/s20123450

**Published:** 2020-06-18

**Authors:** Muhammad Diyan, Bhagya Nathali Silva, Kijun Han

**Affiliations:** School of Computer Science and Engineering, Kyungpook National University, Daegu 41566, Korea; m.diyan@knu.ac.kr (M.D.); nathalis@netopia.knu.ac.kr (B.N.S.)

**Keywords:** reinforcement learning, home energy management, appliance scheduling, human-appliance interaction, user comfort

## Abstract

Maintaining a fair use of energy consumption in smart homes with many household appliances requires sophisticated algorithms working together in real time. Similarly, choosing a proper schedule for appliances operation can be used to reduce inappropriate energy consumption. However, scheduling appliances always depend on the behavior of a smart home user. Thus, modeling human interaction with appliances is needed to design an efficient scheduling algorithm with real-time support. In this regard, we propose a scheduling algorithm based on human appliances interaction in smart homes using reinforcement learning (RL). The proposed scheduling algorithm divides the entire day into various states. In each state, the agents attached to household appliances perform various actions to obtain the highest reward. To adjust the discomfort which arises due to performing inappropriate action, the household appliances are categorized into three groups i.e., (1) adoptable, (2) un-adoptable, (3) manageable. Finally, the proposed system is tested for the energy consumption and discomfort level of the home user against our previous scheduling algorithm based on least slack time phenomenon. The proposed scheme outperforms the Least Slack Time (LST) based scheduling in context of energy consumption and discomfort level of the home user.

## 1. Introduction

The requisition for electrical energy, smart grid paradigm, and renewable energy extend to new space for the home energy management system (HEMS) in such a way that can mitigate the consumption of smart home energy. The HEMS integrates a demand response (DR) mechanism that shifts and restricts demand to improve smart home energy consumption [1]. Normally, this system builds an ideal usage in the views of energy cost, user comfort, load profile, and environmental concerns. During on-peak hours, the DR shifts the load to off peak hours according to price of electrical energy. The smart home automatic energy management control system (HEMS) optimizes the scheduling of household appliances when the cost of electrical energy is high [2]. Therefore, this HEMS schedules the appliances according to the energy consumption and cost, taking account of different parameters i.e., load profile, energy price, weather, and user preference etc. Energy prediction and proper feedback reduce the utility bill by 12% [3]. Moreover, the automation of the home through appliances scheduling and their load profile prediction minimizes the cost of electricity for the user according to the user comfort, reduces the load on the grid, and controls fluctuation in DR, with less human interaction and a high effect for the environment. [4,5]. Intelligent techniques have been used to curtail the price of energy consumption and schedule home devices using real-time monitoring and control, stochastic scheduling, and optimal decision making [6]. A real-time monitoring system schedules the loads according to the load condition and load display of the controllable appliance in real time. The overall cost of energy is computed by using stochastic dynamic programming to control a set of home appliances. Finally, selected devices are controlled and scheduled in real-time control based-HEMS. However, these intelligent algorithms are difficult to operate in an environment with low speed and an outsized computational load.

Artificial intelligence (AI)-based HEMS has received much attention in the past decade, with most of the systems implementing a household appliance scheduler and controller for consumers in smart homes to reduce the energy cost. Further, these systems are based on the adaptive neural fuzzy inference system (ANDIS), fuzzy logic control (FLC), and artificial neural network (ANN) [1]. The AI scheduler and controller is incorporated with a programming code that mocks the human nerves system [7]. ANN consists of input, output, and hidden layers (in some cases) as well as data processing algorithms which model the nonlinearity of the systems and mimic the human brain, put to use as a smart scheduler and controller to schedule smart home appliances [8]. ANN-based controller and scheduling models can be used for prediction and controlling instead of other conventional simulation-based methods to predict and control the cost of electricity. An ANN-based proposed scheme was introduced in [9]. The utmost goal of this scheme was to evaluate the demand load (DL) as a function of demand limit, daytime, price on Time Of Use (TOU), and ambient temperature. A machine learning-based DR approach was developed in [10,11], for circulation of loads mainly focused on home heating, ventilation, and air conditioning (HVACs). For the prediction of different residential load responses to various price signals, an ANN and wavelet transform (WT) approach was in employed in [12,13,14]. The HEMS made the existing home much smarter by enabling consumer participation. Real-time communication between consumer and utility was possible through HEMS. The consumer could adapt their energy consumption on the basis of the price hiked in peek time, comfort level, and also environmental concerns.

Human behavior is complex and hard to predict, which depends on environmental aspects i.e., climate and characteristics of the building [15]. An activity performed by the human-appliances interaction log in the HEMS through the HEMS framework, where the sensors attached to households, provides activity data to the HEMS. After collecting the data from the human-appliances module, the HEMS does many jobs specifically, identifies the number of users and their corresponding actions, user classification according to the living space, forecasts the user load profile to predict the future energy consumption and cost. Furthermore, user behavior plays a vital role in designing home automation that replaces the conventional HEMS with new automatic real-time HEMS [16,17]. The most common example of human–appliances interaction is the smart home environment. In the smart home paradigm, the user plays a main role where the HEMS gets data from human-appliances interaction for (1) data and user classification, (2) scheduling the appliances according to the user preference and electricity cost, (3) providing services to the user. Human-appliances interaction data consist of (a) Time horizon data, weather data, electricity price data, zone wise ambient temperature, and user up-to-date location-based data and actions performed by user contained data, (b) control signal data related to appliances, and (c) indoor and outdoor temperature data, and user comfort preference data about services provided by household appliances [18,19].

Generally, the household appliances are categorized within three major classes based on their features and preferences, including un-adaptable, adaptable, and manageable loads [20,21,22]. The mathematical equations and objective function of the load management, together with the numerous constrained appliances operation for said categories of appliances, are explained in the following sections.

### 1.1. Un-Adaptable Load

Un-adaptable load has strict energy demand requirements that must be satisfied during the load distribution process, e.g., surveillance system, security alarming system, and refrigerators (REFG). Once the un-adaptable load initiates an operation, it is hard to schedule and operate continuously. The energy consumption of this load profile is usually equivalent to the energy demand of this load profile [22]:

(1) $$EC_{k,t}^{un}=ED_{k,t}^{un},$$

where *k* ∈ {1, 2, 3 …*K*} represents an appliance, *K* represents total number of appliances, *t* ∈ {1, 2, 3 …*T*} represents the time in hour, and *T* represents the last hour of the current day, i.e., *T* = 24, assuming that the tariff is revised hourly. $ED_{k,t}^{un}ED_{k,t}^{un}$ and $EC_{k,t}^{un}$ $EC_{k,t}^{un}$ express the load demand and the explicit energy consumption of household *k* at hour *t*, respectively. In this case, the cost of the appliance is equivalent to the total energy consumption of the consumer bill. So, for the un-adaptable appliance *k*, the utility function is:

(2) $$U_{k,t}^{un}=T^{0}EC_{k,t}^{un},$$

where $T^{0}$ expresses the electricity tariff at time *t*.

### 1.2. Adaptable Load

Adaptable load appliances schedule their operation in off-peak hours when the cost is low in the schedule time horizon. However, during the peak hours, if the demand of these devices cannot be satisfied, these appliances shut down automatically and reschedule the incomplete operation in off-peak hours.

During the off-peak hours, the electricity tariff is inexpensive and thus the adaptable load appliances schedule their demand load in these hours. Taking the advantage of off-peak hours not only are peak hours avoided, but also up to the maximum drop off in the electrical bill. Further, the adaptable load has two possible operating states, “Power On” and “Power Off” [22]:

(3) $$EC_{k,t}^{\mathrm{adapt}}=B_{k,t}ED_{k,t}^{\mathrm{adapt}}.$$

For appliance *k*, $B_{k,t}$ represents the binary variable i.e., $B_{k,t}$ = 1, assuming that *k* operates at hour *t*; otherwise, $B_{k,t}$ = 0. Moreover, two forms of costs are derived, in this class of appliance i.e., for the electrical bill and discomfort time for an appliance to start and finish the current operation. For example, the dishwasher (DW) normally operates in period [*T_k, start_, T_k, finish_*], however, the operation time can be scheduled from peak hours to off-peak hours, whenever the DW initiates at *T_k,dw_*, in this scenario, the discomfort waiting period would be *T_k,dw_ − T_k, start_*.

Hence, for the adaptable appliance k, the utility function becomes as follows [23]:

(4) $$U_{k,t}^{\mathrm{adapt}}=T^{0}\;.\;EC_{k,t}^{\mathrm{adapt}}+l_{k\;.\;}\left(T_{k,dw}-T_{k,start}\right),$$

(5) $$T_{k,start}\;\leq T_{k,dw}\;\leq [T_{k,finish}-\;T_{k,ne}],$$

(6) $$T_{k,ne}\;\leq \;T_{k,finish}-T_{k,start},$$

where in the first equation, the first term shows the cost of the electricity and the second term indicates the discomfort waiting time cost. In addition, *l_k_* indicates the system dependent coefficient. Further, *T_k, start,_ T_k, finish_* are the start and finish periods, *T_k,dw_* shows the current operation initiation time, and *T_k, ne_* is the time required for the completion of an operation of the adaptable load.

### 1.3. Manageable Load

Manageable load is totally different from the rest of the two loads and can be operated in manageable power consumption mode between minimum and maximum energy demand and can be donated as $ED_{k.min}$ and $ED_{k.max}$, respectively, when tariff hikes appliances, for example, fan, light, and air conditioner (AC) revise their energy consumption between the $ED_{k.min}$ and $ED_{k.max}$ power range [22]:

(7) $$EC_{k,t}^{mng}=ED_{k,t}^{mng},$$

(8) $$ED_{k.min}\;\leq \;ED_{k,t}^{mng}\leq \;ED_{k.max}.$$

The main purpose of this class of appliances is to minimize the electricity bill and keep the demand low in peak-hour slots, moreover, this situation may lead to the discomfort wait for the consumer. So, the utility function for the manageable load profile appliance k is shown as follows [22]:

(9) $$U_{k,t}^{mng}=T^{0}EC_{k,t}^{mng}+\beta_{k}*{\left(EC_{k,t}^{mng}-ED_{k.max}\right)}^{2}.$$

In the above equation, the first portion exhibits the energy cost, and the last item indicates the user discomfort wait cost, adopted from [24]. The *β_k_* is the appliance dependent discomfort cost parameter. If an appliance has high *β_k_* value, the appliance requires high energy to support the comfort level and vice versa.

### 1.4. Objective Function

The consumer objective function minimizes the electricity cost as well as minimizes the discomfort wait cost and can be expressed as follows:

(10) $$min\overset{k}{\underset{k=1}{\sum }}\overset{t}{\underset{t=0}{\sum }}\{\begin{array}{c} \left(1-\rho \right).T^{0}.\left(EC_{k,t}^{un}+EC_{k,t}^{\mathrm{adapt}}+EC_{k,t}^{mng}\right)\\ +\rho .\left(\begin{array}{c} n_{k}.\left(T_{k,dw}-T_{k,start}\right)\\ +\beta_{k}.{\left(EC_{k,t}^{mng}-ED_{k.max}\right)}^{2} \end{array}\right) \end{array},$$

where the initial part of the equation represents the energy cost, and the last part of the equation denotes the discomfort wait cost. For balancing the electricity and the discomfort wait cost, ρ is used as a tuning parameter [23].

## 2. Related Work

A vast amount of literature on smart home energy control and management has been proposed. For instance, in [25], the authors introduced the innovative predictive and adaptive heat control mechanism based on an ANN in urban smart buildings to enhance and produce suitable consumer thermal comfort environments. The results showed that the ANN-based controller can increase thermal comfort in residential buildings. But they did not consider energy cost and optimization. A game-based approach was introduced in [26] for adjusting electrical energy used by a residential user. In this study, a distributed energy consumption scheduling algorithm was proposed in order to control the cost of energy and balance the load among multiple users, and it reduced Peak-to-Average Ratio (PAR), energy cost, and daily electrical charges of each user. However, the authors did not consider the controlling scheduler and user comfort. Encroachment in HEMS commenced in late 2012, when an AI-based HEMS with DR was urbanized to moderate energy intake and electricity energy cost [27]. In this effort, an intelligent algorithm for controlling appliances and analyzing the DR with simulation study was presented. On the basis of importance and user comfort levels of four households’ appliances, explicitly, air condition, heater, vehicle, and dryer, were managed and controlled. But they failed to add users’ accessibility and environmental concerns for their energy cost module. In [28], Niu-Wu, D et al. designed a hybrid scheme for load-forecasting in Smart Grid (SG). They used a support vector machine and the ant colony optimization-based technique for forecasting DR. In this article, the authors preprocessed the input data through the ant colony optimization technique and for feature selection, the mining technique was used. The particular data features were fed into the support vector machine-based predictor. The authors compared Support Vector Machine (SVM) with the single ANN and validated the proposed scheme effectiveness for short term load forecasting. In this article, the author did not address the demand side satisfaction degree for their efficient forecasting and user comfort. Furthermore, the ANN cum genetic algorithm-based method was used in one study [29]. In this study, home appliances were scheduled with optimized electrical energy consumption in a housing zone to shrink energy demand during peak hours and take full advantage of the usage of renewable energy. However, the authors neglected controlling the user comfort. Additionally, in [30], Anvari-Moghaddam et al. proposed a multi objective mixed integer nonlinear programming design for optimum power consumption in a smart home by considering meaningful stability among power saving and a user comfort zone. The user comfort and power saving were balanced through mixed objective function under user priority and various constraints. This scheme reduced the energy consumption, utility bills, and also ensured user comfort. The renewable energy integration is not addressed in this research study. In [31], a hybrid algorithm was presented based on ANN to reduce home energy consumption and electricity bills by predicting the ON/OFF status of appliances. This scheme guaranteed an ideal control scheduling and reduced peak hour energy consumption. Though, this work did not consider user comfort zone. Yet, there are still challenges in several literature works on smart HEM systems. Much work on the potential of HEMS was carried out in [32,33], in context of the smart grid, an optimization-based method for effective demand side management was presented. The cost reduction issue was formulated for the end user. The price and energy minimization during peak and off-peak hours were handled by scheduling heterogeneous devices i.e., renewable and electrical appliances. Underlining that these methods primarily depend on corresponding existing demand-generation by monitoring and optimization of the energy usage of household appliances at the user end. However, user comfort is ignored in scheduling household appliances in home scenarios. Aram et al. [34] presented an energy conservation methodology by decreasing the amount of necessary communication. This approach utilizes no-linear autoregressive neural networks to predict specific volume of sensed data. The performance of said system is estimated from humidity and temperature data attained from corresponding sensors under various circumstances, specifying that the technique considerably minimizes energy usage for wireless sensor networks. The price and electricity bill minimization are not considered in this work. Lee et al. [35] proposed a smart energy management device which embrace the resident’s activities and living patterns as it is aimed to minimize power consumption of some household appliances, like lights and humidifier. Further, 7.5% of energy was aimed to save by this sensor-based system. Moreover, in reference [36], the authors used ANN based on PSO to increase its performance by selecting the learning rates and best neurons in the hidden layer. A hybrid lightning search algorithm (LSA)-based ANN was proposed in [31], to forecast the optimum status on/off of household appliances. The hybridized technique improves the ANN accuracy. In reference [37], an ANN-based distributed algorithm was proposed to reduce the total electricity cost and delay for power demand by taking accurate energy management decisions. The ANN can effectively monitor and manage power consumption by controlling appliance electricity consumption. In [10], authors proposed an associative scheme that integrated machine learning, optimization, and data structure techniques which result in DR-based HEMS. In this work, a machine learning-based DR approach was developed for circulation of loads mainly focus on HVACs. For the prediction of different residential load responses to various price signals, an ANN and WT approach was employed in [12]. Yu et al. [38] proposed an energy optimization algorithm based on deep deterministic policy gradients (DDPGs) for the home energy management system to reduce the cost without violating the indoor comfortable temperature range. The DDPGs-based system takes action concerning the charging/discharging of Energy Storage System (ESS) and HVAC power consumption, considering the current observation information. Li et al. [39] presented a deep reinforcement learning (DRL) technique to schedule the household appliances, taking into account the user behavior, energy price, and outdoor temperature. The DRL used in this scheme takes care of discrete and continuous power level actions which enable the scheduling of distinct load of appliances. Ruelens et al. [40] proposed a Monte Carlo model-free technique that considers a matric depending on the state-action function value (Q-function). This method predicts a day-ahead schedule of the thermostat of a heat pump. In [41], the authors proposed a hybrid scheme consisting of deep Q-learning and deep policy gradient which enable the scheduling of distinct load of appliances in smart community buildings. The algorithm enables a single agent with an appropriate algorithm to take a series of best actions and solve complex tasks. Another study in [42] proposed a control system to optimize the energy consumption in community buildings. The control system analyzes the energy consumption and cost of appliances using a simulator during the peak and off-peak time horizon and makes aware the user to minimize energy consumption. Therefore, the Petri net (PN) model was used to illustrate the energy consumption strategy for community buildings which ensures the user comfort level based on user preference.

The generic energy consumption by various appliances in a home is presented in Table 1. The values in Table 1 reveal that the energy consumption is high in the case of manually switching off and on the appliances.

**Figure 1:**
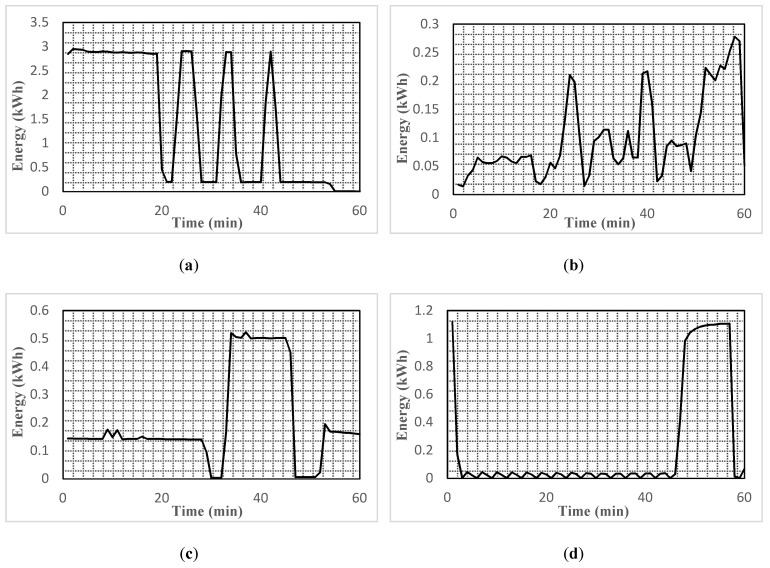

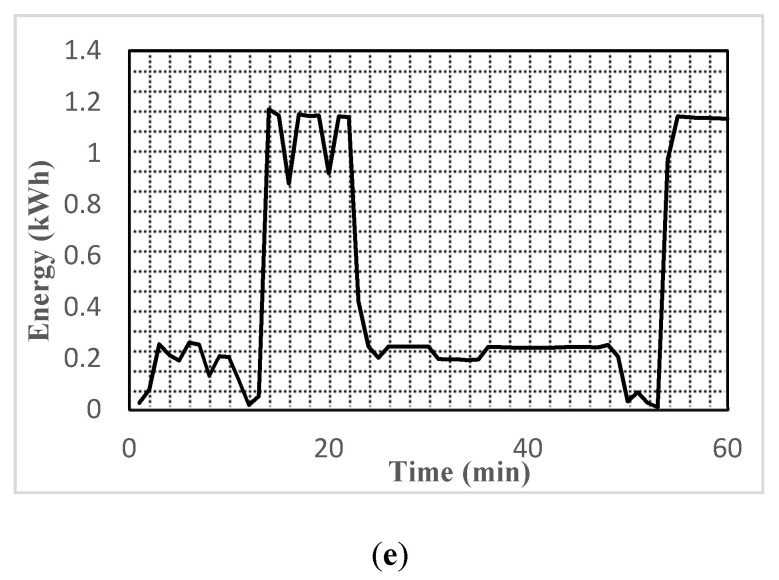
Load profile of various household appliances. (**a**) Energy consumption of dryer; (**b**) energy consumption of washer; (**c**) energy consumption of REFG; (**d**) energy consumption of AC; (**e**) energy consumption of DW [43].

In addition, we examined the energy consumption required by various appliances with an hour time as shown in Figure 1. The graphs show that during switch on time, the appliances required higher energy compared to the rest of the operation. Similarly, some of the devices such as the dryer and washer required higher energy as long as it was operating. The graphs also reveal that during operation of such devices, other devices could be switched off or shifted to an idle mode to optimize energy consumption. However, this could be only performed if we somehow programed the electronic appliances. Moreover, we could tune the working of the rest of the electronic appliances when the REFG and AC was operating or in the peak load time.

Concluding the above literature, we have seen a number of schemes specifically optimizing the energy consumption with renewable energy sources, demand–response-based scheduling of household appliances, traditional scheduling techniques, and machine learning based grid management. However, there is no such system available that models the human-appliances interaction with incorporating the previous appliances data and real-time decision-making system for making the household appliances intelligent. Our aim in this research work is to make the household appliances intelligent in the context of consuming energy and satisfying the comfort level of home users.

## 3. Problem Statement

### 3.1. Motivation

Recently, researchers put a lot of effort in optimizing energy consumption of household appliances. However, there still exists room for improvement of such systems. In addition, such systems consists of a number of challenges, for instance, the conventional HEMS defines the household appliances scheduling as a model-based scheduling problem, where the HEMS application and energy optimization technique require a model. A difficult step in designing a model-based HEMS is to select a proper model and explicit parameters. Considering the complex and unpredicted behavior of the user, proper model and parameters selection becomes more critical. Moreover, the distinct user will expect for the distinct model with distinct model parameters. However, model-based HEMS implementation requires a reliable and efficient approach to determine proper model and related parameters. In addition, current household appliances still require intelligence to share their current energy consumption state and other relevant parameters with the rest of the household appliances available in a smart home. However, getting the households intelligent, machine learning algorithms are required. Therefore, employing machine learning algorithms to household appliances to get them intelligent requires specific knowledge and the use of agent-based communication. In general, the current literature consists of several challenges such as:

- Lack of appropriate machine learning implementation at the smart home level;
- High monetary and billing cost of implementation;
- High energy consumption due to inappropriate scheduling of household appliances;
- Inappropriate human-appliances interaction;
- Intelligent communication network among smart homes and smart grids;
- Modeling the unexpected behavior of humans in operating the smart home appliances;
- Irregular utilization of household appliances;
- Inadequate consumer comfort;
- Modeling the operation of appliances along the day-time horizon;
- Demand-response-based scheduling does not guarantee the low energy consumption;
- Wireless Sensor Networks (WSN) based smart home energy management systems.

### 3.2. Contribution

The current research literature targets specific scenarios, specific range of appliances, complex design, etc. In addition, the traditional methods are mostly based on time consuming algorithms and processes which ultimately give inappropriate decisions with new appliances and complex systems (smart grid). One of the major reasons of failure of such systems is the difficulty to model human behavior toward electronic appliances. For instance, one can create many user profiles based on the previous information of a single home user. Similarly, if there are several users in a home, then it would become impossible to appropriately manage user profiles and process it through algorithms based on neural networks or machine learning techniques. Further, if somehow, we succeeded in modeling human behavior toward electronic appliances, then it may be easy to optimally schedule the electronic appliances in a smart home. In this research work, we propose a scheduling algorithm based on human-appliances interaction in smart homes using the Reinforcement Learning (RL) algorithm. The RL comes up with the best scheduling strategy for minimizing the household appliances energy consumption while offering minimum discomfort level.

The main contributions of this research work are as follows:

(a) Though, there is no such research studies available till this day providing smart home appliances with intelligence. This research put forward an idea of making the smart home appliances intelligent with the reinforcement learning. The household appliances are made intelligent and, therefore, they can decide intelligently whenever the energy consumption of the smart home exceeds a certain limit. They also can share their status such as priority information of households, status, etc. with other appliances.
(b) A new RL-based energy management and recommendation system (EMRS) is proposed that enables smart home appliances to consume energy through the optimal scheduling of appliances. In EMRS, a reinforcement learning algorithm called Q-learning is used to schedule the energy consumption of different appliances. Whereby, the Q-learning algorithm attaches agents to each household appliance and determines an optimal policy to reduce the energy consumption and electricity billing without disruption of user comfort level.
(c) A discomfort function is introduced to model the discomfort and arises due to scheduling the household appliances against the energy consumption.
(d) Finally, the proposed is tested on households against the TOU pricing tariff strategy. The proposed system efficiently reduces the energy consumption and discomfort of the home user. On the other hand, the proposed system is compared with the scheduling algorithm based on the LST algorithm. The results reveal that the proposed system outperforms the LST-based scheduling in context of energy consumption and user discomfort of the smart home user.

## 4. Proposed Scheme

### 4.1. Birdseye View of the Proposed Scheme

**Figure 2:**
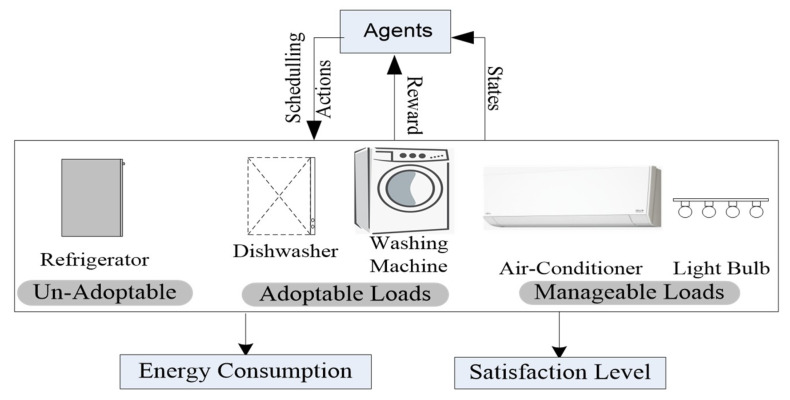
Birdseye overview of the proposed scheme.

Much work on the potential of standardizing HEMS has been carried out, unfortunately, there are still some critical issues which draw our attention. Moreover, in the last decade, we have observed that very few studies have been published in this regard. Accordingly, to go a step ahead of designing specific energy optimization methods and algorithms for specific scenarios, we come up with an intelligent automatic energy management and recommendation system to interconnect user and smart home objects of various load profiles such as adaptable, unadaptable, and manageable as shown in Figure 2. In our proposed architecture, we presented an automatic energy management and recommendation system (EMRS), wherein user and different class of household appliances are equipped with one EMRS, with the concerns to reduce their electricity consumption and user discomfort, as shown in Figure 1. Human-appliances interaction (HAI) that enables communication and exchange of human-appliances interaction information pattern and energy consumption of appliances. EMRS receives the human-appliances interaction information log from the user activity model, and then in response to the user behavior, the EMRS schedules each appliance to the evaluated user profile. In this work, we integrate the EMRS with the Markov decision process (MDP) and use the reinforcement learning-based method called Q-learning to model the HAI and schedule different home appliances (REFG, TV, bulb, AC, WM, DW) and recommend an optimal scheduled sequence of appliances to the user according to the HAI model. In the Q-learning paradigm, the agents interact with the environment and learn to take an optimal action based on their current state. A simple reward from the environment is set to motivate the agents. From the set of different states, the agent obtains an optimal policy. Eventually, to maximize the reward, each agent acquires knowledge to choose the best action. It is accomplished by the agent to measure the current state value, which will be precise by visiting the next state. The action is picked, which maximizes reward value of the next state. In this work, each appliance has their own single Q-learning agent, the agent learns and exhibits a sequence of optimal policy based on the HAI model of the user for operating the appliances to overcome the power consumption rate and user discomfort level. Each appliance is modeled as an environment with state and aspect. The user interacts with the appliances to enable an operation for the user, in response, the appliances execute the operation and give the required service and comfort to the user. When an appliances agent is participating in the energy management control system to reduce the power consumption and cost, then the agent will have an automatic and recommended policy for turning off specific appliances which can create greater discomfort to the user. During the learning process, the HAI model gives random action feedback to the agent and this feedback comes from the user comfort value of the state when switching off that specific appliances. The calculation of the comfort level is based on the user preferences and power consumption which measure the user–appliances interaction at that time. The user comfort level may change occasionally due to some circumstance i.e., such that user routine, weather, and emergency situations.

### 4.2. Q-Learning-Based Propsoed Emrs Model

While employing the reinforcement learning in the proposed model, the smart home energy environment is considered an MDP problem. The MDP environment consist of five elements (S, A, P, R, *γ*), S represent the set of finite discrete states in the MDP environment, where A is the available set of actions for an agent, P denotes the probability matrix of the state transition, R represents the reward, and *γ* denotes a discount factor amid 0 < *γ* > 1 and utilization for the relation of the current versus future reward. An agent in RL continuously reaches out to the environment and picks an optimal policy by visiting almost every state. When an agent interacts with the environment at every observation, the agent reaches a state *s_t_* ∈ *S* in time *t*. Accordingly, the agent chooses and performs an action *a_t_*. Afterwards, the current state of the agent environment will be transferred to state *s_t_*_+1_ ∈ *S* by considering the P (transition probability matrix). Thus, the agent gets an on-hand reward *r_t_* by considering the R (reward function). If the taken action leads to a promising environment reward, then the attraction of this action-reward pair will be given preference, and vice versa. The primary goal of the agent to maximize the collective discounted reward at time *t* as the following:

(11) $$R_{t}=\overset{\infty }{\underset{k=t}{\sum }}\gamma^{k-t}r_{k\;}=r_{t\;}+\gamma R_{t+1}.$$

It is useless to get all the rewards and calculate the discount rewards for every state. To overcome this limitation, a model-free RL method is introduced in which Q-function is used at state ‘s’ to calculate the best value and select a proper action *a_t_*. Specifically, the Q-function is the combined prediction of the cumulative, expected, and discounted future reward and can be computed using Equation (11):

(12) $$Q\left(s_{t},a_{t}\right)=E[r_{t}+\gamma r_{t+1}+\gamma^{2}r_{t+2}+\ldots \ldots ..|s_{t},a_{t}].$$

Q-learning is the effective RL model-free algorithm, instead of the building environment model, the algorithm estimates the action a value at state ‘s’ of the environment. The agent interacts with the environment and takes an estimated action. From the environment, the agent receives a new state with the reward for the environment. The process is suspended once the agent maximize the rewards. A policy is determined from the agent taken action in the specific state; hence, the objective of the agent is to find an optimal policy which maximizes the reward. For these kinds of decision making problems, Q-learning is the suitable method for finding and selecting the optimal policy v. Further, the Q-learning method calculates $Q\left(s_{t},a_{t}\right)$ pair and updates the Q-value against the cumulative rewards considering the below Bellman equation [44]:

(13) $$Q_{t+1}\left(s_{t},a_{t}\right)=Q_{t}\left(s_{t},a_{t}\right)+\alpha \left[y_{t}-Q_{t}\left(s_{t},a_{t}\right)\right],$$

(14) $$y_{t}=r_{t\;}+\gamma maxQ_{t}\left(s_{t+1},a^{\prime }\right),$$

(15) $$Q_{v}\left(s_{t},a_{t}\right)=r\left(s_{t},a_{t}\right)+\gamma maxQ\left(s_{t+1},a_{t+1}\right),$$

Where *y_t_* represents the desirable value, as from the beginning it is anonymous. The agent calculates the approximation of the desirable value i.e., *y_t_* in Equation (13) from the immediate reward and maximum Q value of the next state. In Equation (14), the pair addition of maximum discount factor $\gamma maxQ\left(s_{t+1},a_{t+1}\right)$ and the current reward $r\left(s_{t},a_{t}\right)$ give the optimal Q-value $Q_{v}\left(s_{t},a_{t}\right)$ in context of optimal policy ‘v’. *γ* denotes a discount factor amid *γ* ∈ (0, 1) and utilization for the relationship of the current versus future reward. By decreasing the discounting factor *γ*, the agent picks the present immediate reward and when the ‘*γ*’ is increasing and closet to 1, the agent tends to consider the future reward. When an action takes place in state ‘s’ at time *t*, the Q-value is altered in Q-table. From the same table, the agent selects the future actions in time *t*, and by using the following Bellman equation, the agent updates the selected state-action pair value in the Q-table:

(16) $$Q_{t+1}\left(s_{t},a_{t}\right)=\left(1-\theta \right)Q_{t}\left(s_{t},a_{t}\right)+\theta \left[r\left(s_{t},a_{t}\right)+\gamma maxQ\left(s_{t+1},a_{t+1}\right)\right].$$

In Equation (14), *θ* amid [0, 1], which denotes the learning rate of the agent by receiving the observation (trail-error) from the environment up to the extent where the value of Q-table is altered. When the *θ* value is near ‘0’, the agent picks the previous Q-value and learns nothing and uses exploitation in the Q-learning process. Moreover, when the *θ* value is near ‘1’, the agent picks the present reward and maximizes the future discounted reward by using the exploration strategy in the Q-learning process. Like the *γ*, the system operator can set the value of *θ* in between ‘0’ and ‘1’to ensure the exploitation and exploration using Equation (15), while updating the $Q_{t}\left(s_{t},a_{t}\right)$ in an iterative mode. At certain time *t*, progressively the Q-value will reach a larger value, and using the following equation with larger Q-value, the agent will get the projected optimal policy v:

(17) $$v=\mathrm{argmax}Q\left(s_{t},a_{t}\right).$$

In this research, the abovementioned RL-based Q-learning model-free method is used where each appliance acts as a Q-learning environment with an agent to determine an optimal policy for operation of appliances. Our proposed work targets the consumer electricity consumption plus consumer comfort level by scheduling preferred appliances. The following section describes the components of the proposed Q-learning i.e., states, actions, and rewards in context of smart home energy management and the recommendation control system.

#### 4.2.1. States

In context of smart home energy management and the recommendation control system, in a house, each appliance refers to the Q-learning environment and the associated load profile or the power rating is called the state. In the Q-learning paradigm, an environment should have one or more than one state, in smart home energy management and the recommendation control system, an agent has more than one goal state by performing couple of actions. In our system, a goal state can reach once the current energy consumption is less than or equal to the available energy in the current state the agent can consume. Further, in our proposed system, we have three classes of appliances i.e., (1) un-adaptable load (2) adaptable load (3) adaptable load. From each class of appliances, we pick one or more appliance with their corresponding load profile, namely REFG, WM, air-conditioning system (AC) and light1 (L1), light2 (L2) with the load profile as shown in Table 2. The total power for a house is considered to be 2500 watts. The agent compares the current power consumption with the available energy, the goal of the smart home agent is to keep the power consumption less than that of the available energy. The agent does nothing when the current energy consumption is fewer than the available energy and can turn on another preferred appliance. The states of the different classes of appliances are given below:

(18) $$S^{\mathrm{REFG}}=E^{\mathrm{REFG}}\;,S^{\mathrm{WM}}=E^{WM},\;S^{\mathrm{AC}}=E^{\mathrm{Ac}}.$$

In Equation (17), $E^{\mathrm{REFG}}\;,\;E^{WM},\;and\;E^{\mathrm{Ac}}$ represent the states which is actually the power rating of the REFG, WM, and AC at time *t* [45].

#### 4.2.2. Actions

In the proposed Q-learning for the smart home energy management system, the action for an appliance varies from state to state in the agent environment. In this work, we have various actions for the said three classes of appliances i.e., for (1) un-adaptable load, the agent has no action to perform, (2) adaptable load, the agent has two actions ‘On’ and ‘Off’, and (3) adaptable load, the agent has ‘n’ level of actions. The action of different classes of appliances are stated below [45]:

(19) $$A^{REFG}=\left\{On\right\}\;OR\;A^{REFG}\neq \left\{Off\right\},$$

(20) $$A^{WM,DW}=\{On\;OR\;off\},$$

(21) $$A^{AC}=\{0,\Delta E^{\mathrm{Ac}},2\Delta E^{\mathrm{Ac}},3\Delta E^{\mathrm{Ac}},\ldots ,8\Delta E^{\mathrm{Ac}},9\Delta E^{\mathrm{Ac}}\},$$

(22) $$A^{L1,L2}=\{0,\Delta E^{L1,L2},2\Delta E^{L1,L2},3\Delta E^{L1,L2},\ldots ,6\Delta E^{L1,L2},7\Delta E^{L1,L2}\}.$$

In Equation (18), the REFG agent will perform only on action. In Equation (19), the WM and DW agent will perform one action i.e., switch on whenever the agent turns on the WM, the constant energy consumption starts *E^WM^*, *E^DW^* and when the operation cycle completes, the devices switch off automatically and the agent updates its corresponding record. In Equations (20) and (21), the AC and light bulbs (L1, L2) have ten and eight discreet energy levels available respectively, which means the agent can take 10 actions for AC and 8 actions for L1 and L2 to manage the operations in certain conditions. The AC and L1 and L2 energy consumption are represented by Δ*E*^AC^ and $\Delta E^{L1,L2},$ recpectively.

#### 4.2.3. Rewards

In the proposed scheme, we calculate the reward based on the agent actions and appliances priority. We define the reward matrix based on user preference as given below:

Reward={(23)−1  when the goal state energy exceeding from the avialable energy(24)0   Do nothing(25)1   when the goal state reached after turning off an appliance

#### 4.2.4. Discomfort Level

**Figure 3:**
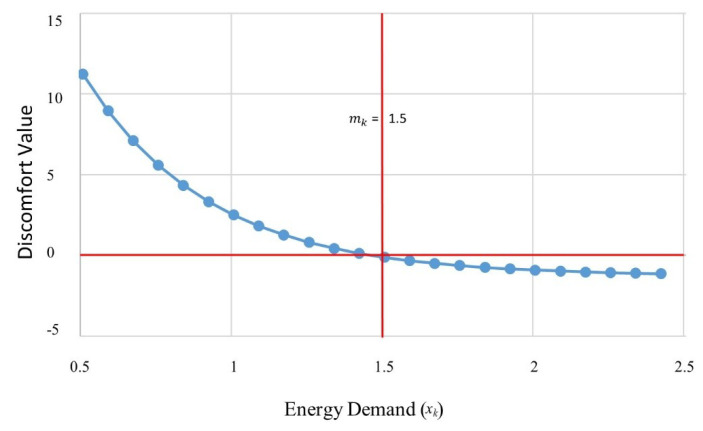
Discomfort changes against energy demand.

In the proposed work, we calculate the user discomfort cost against the energy consumption of all the household appliances [46]:

(26) $$\varphi_{k}\left(x_{k}\right)=e^{\beta \left(1-\left(\frac{x_{k}}{ED_{k}}\right)\right)}\;-\;1,\;\beta k>0,$$

where K represents the set of appliances and |K| = N, for each appliance, k ∈ K and *x_k_* represent its strategy. We model the set of appliances ‘K’ latter cost $\varphi_{k}\left(x_{k}\right)$ as a discomfort cost function. Generally, $\varphi_{k}\left(x_{k}\right)$ with respect to *x_k_* the function value is continuously decreasing from positive to negative at the median level of the energy demand (represented by $m_{k})$. According to [47], this function has three fundamental properties. (1) If *x_k_* is less than $m_{k}$, meaning that the discomfort value is positive and the appliance is not satisfied with the current demand. (2) If *x_k_* is greater than $m_{k}$, meaning that the appliance is satisfied with the current demand and the discomfort value is positive. (3) When *x_k_* $x_{k}$ equals to $m_{k}$, meaning that the appliance shows neutral behavior to the current energy demand as shown in Figure 3. For achieving these properties, Equation (25) was used where *β_k_* denotes the appliance priority factor. An appliance with the higher *β_k_* has low priority of energy demand and vice versa. Specifically, the appliance, which closely affects the user comfort level, has smaller *β_k_* value [46].

## 5. Experimental Analysis

### 5.1. Simulation Setup

We considered a smart home environment with three major classes of loads adoptable, un-adoptable, and manageable. The performance of our proposed Q-learning algorithm is evaluated using Python programming language. We conducted our simulation on six household appliances i.e., two adoptable loads (WM, DW), one un-adoptable (REFG), and three manageable load (AC, L1, L2). The household appliances are randomly turned on and off during the entire course of the day. Similarly, modeling human–appliance interaction exhibits a random nature and, therefore, hard to predict. According to the human-appliances interaction, the user could have different requirements in various situations, in context of power requirement, location, weather, and time. With regard to multiple conditions, for example, during the day time when outside is sunny, high temperature, then the requirement for AC and Ls depends on the position of the user in the house (bedroom, drawing room). In the simulation, we have considered the discomfort parameter *β* depending on the nature or type of the household appliances. Finally, all the simulation parameters are listed in Table 2 [22]. 

Furthermore, the energy data of the household appliances and TOU tariff according to Table 3 is provided to the HEMRS. Resulting, the HEMRS is enabled to take optimal actions by considering the tariff, load priority, and user comfort level. One of the main advantages of the HEMRS is shifting the load of low priority appliances during the peak hours’ time of the day. While updating the Q-table, the agent of each appliance visits all the states and learns new knowledge from the environment. To enhance the learning process of the Q learning, initially, the exploration and exploitation parameter ε is set to 0.2. In addition, the discount factor *γ* is set to 0.9 to update the $Q\left(s_{t},a_{t}\right)$ during error and trail experiences from Q-learning episodes. The learning rate of the system *θ* is set to 0.1.

### 5.2. Results and Discussion

**Figure 4:**
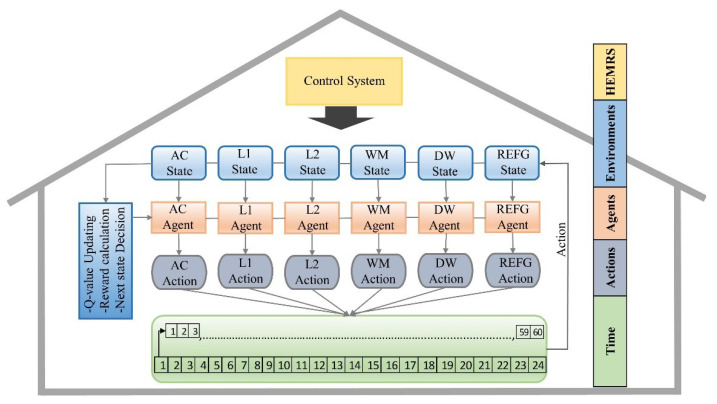
Proposed Q-learning simulation setup.

**Figure 5:**
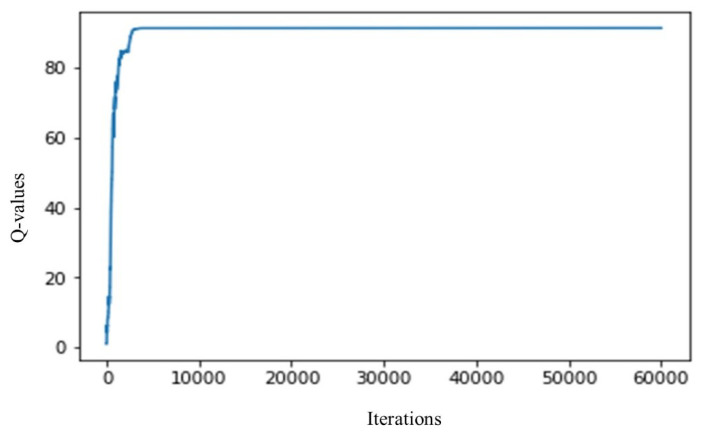
Convergence of the Q-value.

Subsequently, executing the Q-learning simulation setup as shown in Figure 4, the agent starts taking actions randomly. Initially, the agents pick expensive actions, which results in poor Q-value. During this trial and error phase, followed by the successive iteration, the agents explore the available states and actions which results in maximum Q- value as shown in Figure 5. In the proposed scheme, the agent can get high Q-value by performing switching off actions. The switching off actions produce high reward and maximize the Q-value. Simultaneously, the Q-learning algorithm minimizes the monetary cost by keeping the power consumption levels of a household appliance less or equal to the available power. The simulation time of all household appliances was carried out for 24 h. In this section, we compute the throughput in terms of power consumption, monitory cost, and user discomfort reduction. The results obtained from RL based Q-learning algorithms for consumption, monitory cost, and user discomfort are discussed in the following sections. The proposed scheme is compared with one of our previous schemes presented in [48]. In [48], the authors proposed a load balancing and appliance scheduling based on automated switching off system and least slack time algorithm, respectively. However, the approach proposed in [48], does not address the problems i.e., load balancing, scheduling of household appliances, and house hold discomfort level in the proper way. Moreover, during the load balancing based scheduling, the scheme in [35] automatically turns off an appliance without knowing the user need, resulting in high discomfort level. In addition, the scheme in [35] has high energy consumption, high monetary cost, and high discomfort level. Henceforth, to overcome the problem in LST-based scheduling, we proposed the RL-based scheme, which minimizes energy consumption, monetary cost, and user discomfort level by incorporating an intelligent system of performing those actions which results in a high reward.

**Figure 6:**
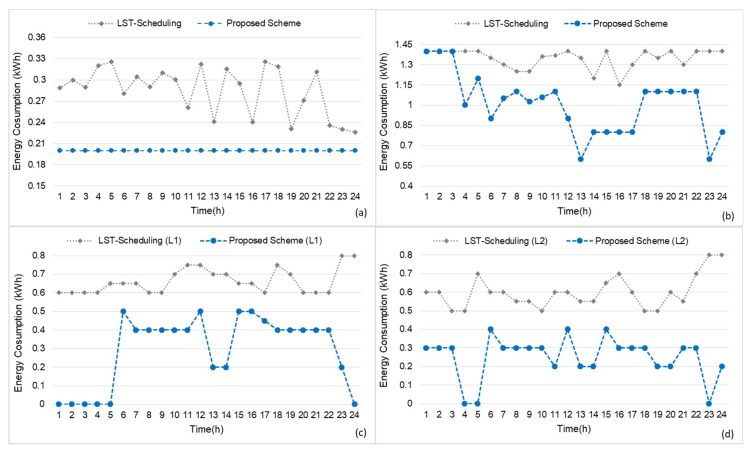
Household energy consumption comparison with LST-scheduling scheme. (**a**) REFG results; (**b**) AC results; (**c**) L1 results; (**d**) L2 results.

The proposed scheme is tested and compared to the home energy management scheme in [48]. As shown in Figure 6a–d, we investigated the energy consumption of REFG, AC, (L1), and (L2), respectively. It was found that the proposed Q-learning-based scheme reduces the energy consumption of each appliance by keeping the total energy consumption less than the available energy and avoids the unnecessary power wastage. In Figure 6a, the results show the performance of the un-adoptable appliance, whereas the energy consumption in our scheme is constant, which means that our scheme kept the switch on the un-adoptable appliance (REGR) for 24 h and consumed less energy during the on-peak hours with less user discomfort. The result shows the average energy consumption of the REFG. On the other hand, during the hypothesis, we investigate that the scheme in [48], sometime automatically switches off the REFG for 10–30 min, which may result in food spoiling. Thus, it highly increases the discomfort of the home user. Moreover, in the case of LST-based scheduling, REFG consumes high energy during on-peak hours. Therefore, it results in high energy monetary cost. Figure 6b–d shows the manageable appliances results (AC, L1, L2), where the proposed scheme schedules and controls the power consumption of appliances with less energy and cost.

The AC results in Figure 6b show that during the on-peak hours, the proposed scheme keeps both the energy consumption and discomfort lower than that of LST-based scheduling, as well as keeps the total power consumption less than the available power. While the results of L1 and L2 in Figure 6c, and Figure 6d, respectively, show that during the nighttime L1 and L2 were scheduled to switch off from 1–5 and 4–5 am respectively. While during daytime and in on-peak hours, the power rating of lights were scheduled low to keep the total power consumption less than the available power. In contrast, L1 and L2 in LST-based scheduling were switched on during the day and night with high power ratings and in on-peak hours, resulting in high energy consumption and cost.

**Figure 7:**
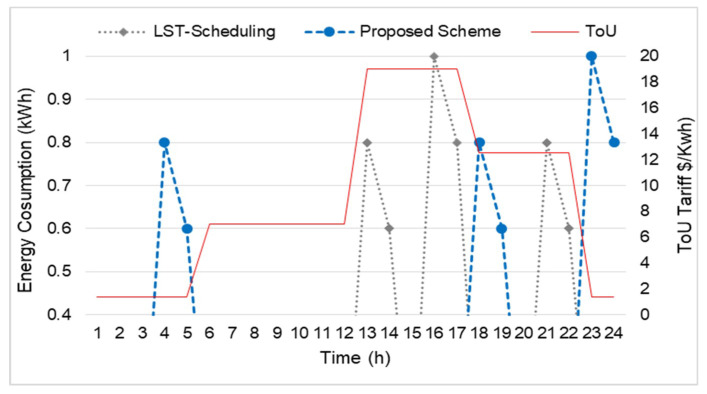
Energy consumption of WM, DW with LST-scheduling.

After getting the maximum Q-value, the energy consumption of the adoptable load can be determined. In Figure 7, the energy consumption of WM and DW of the proposed scheme with the scheme LST-based scheduling is presented. When the proposed Q-learning algorithm deployed, we determined two adoptable appliances i.e., WM and DW, operate and consume energy when the prices are low (overnight hours, off-peak hours, and partial peak hours) and avoids consumption in om-peak time. Specifically, WM consumes energy at time slots 23–24, while, the DW consumes energy during time slots 4–5 and 18–19. During these time slots, the proposed scheme keeps the REFG, AC, L1, and L2 switched the on mode in the low power rating to reduce the user discomfort level. On the other hand, the results reveals that the LST-based scheduling consumes high energy during the on-peak and partial peak hours at time slots 16–17,13–14, and 21–22 in the case of WM and DW. During these time slots, the LST-based scheduling automatically switches off the unadoptable and manageable appliance, which results in a high user discomfort level. The decrease in energy consumption against LST-based scheduling reveals that the proposed scheme can be used in future smart homes.

**Figure 8:**
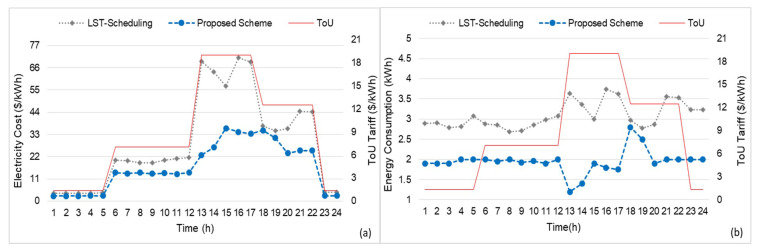
Total energy cost and total energy consumption comparison with LST-scheduling: (**a**) total energy cost of all appliances and (**b**) total energy consumption of all appliances.

In the case of Figure 8, the proposed Q-learning-based scheme is compared with the LST-based scheduling, we figure out the total energy consumption and cost of all appliances. The simulation was 24-h long and depicts the overnight, off-peak, on-peak, and partial peak hours accordingly. Figure 8a clearly reveals that the proposed RL-based Q-learning algorithm significantly minimizes the on-peak load, which reduced the total cost of a smart home. The cost optimization threshold values (available energy) dramatically transferred the on-peak and partial-peak to the off-peak hours with the aid of the Stackelberg game-based dis-satisfaction component [46]. In contrast, the LST-based scheduling has a high energy cost for household appliances in the same duration of time, because it manages its services based on the automatic off option, which leads to higher energy consumption than the proposed scheme during on-peak hours. Figure 8b shows the energy consumption of the proposed scheme against LST-based scheduling of household appliances. As presented in Figure 8b, the proposed Q-learning algorithm-based scheduling has minimized the total power consumption of the household appliances in the on-peak hours and partial-peak hours, as compared to the LST-based scheduling. The LST-based scheduling is highly suitable in scenarios where there is variation in energy consumption of multiple smart home appliances. However, the proposed scheme based on Q learning has the advantages of attaching agents to home appliances, which increases the efficiency by deciding in real time. Similarly, the LST and similar scheduling strategies such as demand-response always perform inappropriate in performing actions in real time.

**Figure 9:**
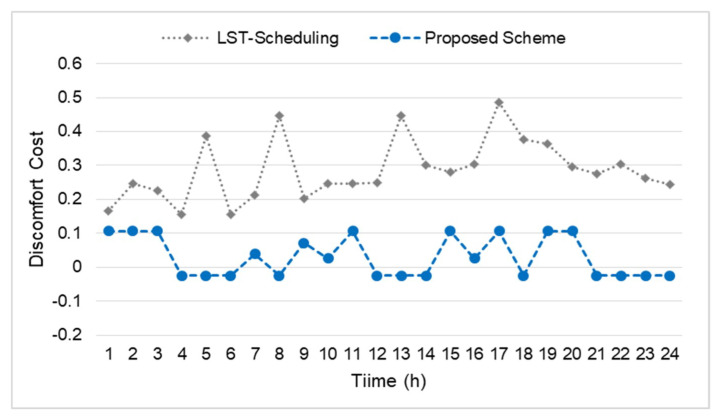
Discomfort level comparison with the LST scheduling scheme.

The user discomfort always depends on the energy consumption. For instance, the higher the energy consumption, the less the discomfort of the home user. Keeping such intentions in mind, Figure 9 shows the user discomfort level compared to the LST based scheduling scheme. The result reveals that the user discomfort level throughout the day is less than that of LST-scheduling. This is due to the energy demand of the appliances of the proposed scheme during the on-peak time being less and high in off-peak hours. In particular, during the on-peak hours, the agent reduces the power level of all that appliances which demanded more electricity to keep the total energy consumption below than that of the threshold energy value and had smaller discomfort *β_k_* value. Contrast to the LST-scheduling scheme, automatically switching off random appliances in higher energy consumption slots and on-peak hours due to the user discomfort level is high in time slot 5, 8, 13, and 17. This experiment reveals that the agents attached to each appliance always learn from the environment and whenever an action is increasing the discomfort of the home user, those actions are always avoided next time. This behavior of the proposed scheme gives high advantage over other similar systems. Finally, the existing literature covers techniques and methods to reduce energy consumption, but they highly increased the discomfort level of the home user. In addition, they never come up with the level of the discomfort due to reduced energy consumption. Keeping a balance equilibrium between energy consumption and discomfort is also one of the main advantages of the proposed scheme.

## 6. Conclusions

In this article, we proposed the scheduling of household appliances based on the well-known reinforcement learning algorithm called Q-learning. The Q-learning algorithm attaches agents to each household appliance. The agents monitor the operation of each appliance and also schedule the operating time of each appliance. In addition, the agents always perform those actions, which increases the rewards i.e., minimum energy consumption. The appliances are divided into three groups i.e., (1) adoptable, (2) un-adoptable, (3) manageable to minimize the discomfort caused by inappropriate scheduling. The proposed system is tested in a smart home environment with a single home user and a number of household appliances for 24 h a day. As we can see, after applying the proposed system the household appliances work intelligently. Therefore, the proposed system efficiently reduces the energy consumption and discomfort of the home user. On the other hand, the proposed system is compared with the scheduling algorithm based on the LST algorithm. The results reveal that the proposed system outperforms the LST-based scheduling in context of energy consumption and user discomfort of the smart home user.

## Figures and Tables

**Table 1:** Load profile of various household appliances [43].

Appliance	Operating Cycles	Operation Load Rang (kW)	Energy Consumption Per Cycle (kWh)	Total Operation Time (min)
DW	Three	0.6~1.2	1.44	105
Washing Machine (WM) and Dryer	Three	0.65~0.52 0.19~2.97	2.68	45+60
REFG	24 h	0~0.37	3.43	24 h
AC	24 h	0.25~2.75	31.15	24 h

**Table 2:** Household appliances simulation parameters.

Device Type	ID	*T_K_*	*β_k_*	Load Profile (Kwh)	Operation Time	*L_n,ne_*
Adoptable	WM	0.1	-	0.52–0.65	6 pm–11 pm	45
	DW	0.1	-	0.6–1.2	6 am–11 am	105
Un-adoptable	REFG	-	-	0.2	24 h	-
Manageable	AC	-	2.3	0–1.4	24 h	-
	L1	-	2	0.2–0.8	6 pm–11 pm	-
	L2	-	2.5	0.2–0.8	6 pm–11 pm	-

**Table 3:** Standard TOU plan with price (cents/kWh).

TOU Plan	Time	Price
Overnight	11 p.m.–5 a.m.	1.34 cents/kWh
Off-Peak	6 a.m.–12 p.m.	7.04 cents/kWh
On-Peak	1 p.m.–5 p.m.	19.01 cents/kWh
Partial-Peak	6 p.m.–10 p.m.	12.50 cents/kWh
